# A Comparative Analysis of Cardiovascular Events Associated With Acalabrutinib Versus Ibrutinib in Chronic Lymphocytic Leukemia: Insights From a Global Federated Network

**DOI:** 10.1002/prp2.70113

**Published:** 2025-05-07

**Authors:** Abdulrahman Majrashi, Ying X. Gue, Alena Shantsila, Stella Williams, Catrin Tudur Smith, Gregory Y. H. Lip, Andrew R. Pettitt

**Affiliations:** ^1^ Liverpool Centre for Cardiovascular Science at University of Liverpool Liverpool John Moores University and Liverpool Heart & Chest Hospital Liverpool UK; ^2^ Department of Emergency Medical Services, College of Nursing & Health Sciences Jazan University Jazan Saudi Arabia; ^3^ Department of Cardiovascular and Metabolic Medicine, Institute of Life Course and Medical Sciences University of Liverpool Liverpool UK; ^4^ Clatterbridge Cancer Centre NHS Foundation Trust Liverpool UK; ^5^ Department of Health Data Science University of Liverpool Liverpool UK; ^6^ Department of Clinical Medicine, Danish Centre for Health Services Research Aalborg University Aalborg Denmark; ^7^ Department of Molecular & Clinical Cancer Medicine University of Liverpool Liverpool UK

**Keywords:** acalabrutinib, atrial fibrillation, cardiovascular toxicities, hypertension, ibrutinib

## Abstract

Chronic lymphocytic leukemia (CLL) is the most common form of leukemia in adults, characterized by the accumulation of dysfunctional lymphocytes. Ibrutinib, a first‐generation Bruton tyrosine kinase (BTK) inhibitor, has significantly improved CLL treatment but is associated with adverse cardiovascular events such as atrial fibrillation (AF) and hypertension (HTN). Second‐generation BTK inhibitors (BTKi) such as acalabrutinib were developed to have greater selectivity for BTK to reduce off‐target effects and improve safety. Comparative real‐world data between ibrutinib and second‐generation BTKi are limited. This study analyzed data from the TriNetX Global Collaborative Network to compare cardiovascular outcomes in CLL patients who received ibrutinib or acalabrutinib. The two groups were well‐balanced using propensity score matching. The outcomes examined included new‐onset AF, HTN, reported heart failure, ventricular arrhythmias, bleeding, and all‐cause mortality. The incidence of AF/flutter was lower for acalabrutinib compared to ibrutinib [5.8% vs. 11.7%; HR 0.59 (95% CI 0.43–0.83); *p* = 0.002]. The incidence of HTN was also lower in the acalabrutinib cohort [15% vs. 26.3%; HR 0.65 (95% CI 0.53–0.81); *p* < 0.05]. The incidence of heart failure, ventricular arrhythmia, bleeding events, or all‐cause mortality did not differ after 3 years of treatment with acalabrutinib or ibrutinib, respectively. Our findings indicate that acalabrutinib has a more favorable cardiovascular toxicity profile compared to ibrutinib; therefore, validating the ELEVATE‐RR trial in a real‐world setting.

## Graphical abstract

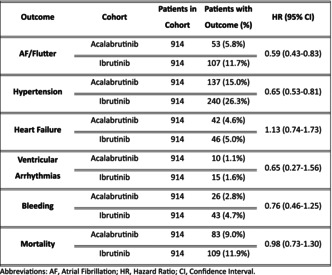

## 1 Introduction

In Western countries, chronic lymphocytic leukemia (CLL) and its noncirculating counterpart, small lymphocytic lymphoma (SLL), are the most common forms of leukemia in adults, with 4–6 cases per 100 000 individuals diagnosed every year, and patients aged 65 or older representing more than 70% of incident cases [1]. Bruton's tyrosine kinase (BTK) is key for the survival and growth of the malignant B cells in CLL/SLL, and the development of BTK inhibitors has greatly improved CLL therapy [2]. Ibrutinib was the first BTK inhibitor (BTKi) to be developed and approved for the treatment of CLL. It blocks the enzymatic activity of BTK by binding covalently to its ATP‐binding domain [3]. Phase 3 clinical trials comparing ibrutinib with chemotherapy or chemoimmunotherapy in previously untreated or relapsed/refractory CLL have shown greatly superior long‐term outcomes in favor of ibrutinib [4, 5]. Although ibrutinib is mostly well tolerated, there are growing concerns about its propensity to cause bleeding and certain cardiovascular adverse events. A meta‐analysis of eight RCTs comparing ibrutinib to chemo(immuno)therapy demonstrated that the risk of atrial fibrillation (AF) and hypertension (HTN) was significantly increased in patients receiving ibrutinib compared to chemo(immuno)therapy [6], while there are additional concerns about heart failure and ventricular arrhythmias [7]. These observations are especially important given the increased prevalence of AF and other forms of cardiovascular disease among cancer patients compared to the general population [8]. The second‐generation covalent BTKi, acalabrutinib and zanubrutinib, have a greater selectivity for BTK compared to ibrutinib [3, 9] and have been compared to chemoimmunotherapy and ibrutinib in phase III RCTs. Acalabrutinib was shown to be superior to chemoimmunotherapy in untreated (ELEVATE TN) and relapsed/refractory (ASCEND) CLL [10, 11], and as effective as ibrutinib but with lower rates of AF and HTN in relapsed/refractory CLL (ELEVATE RR) [12]. More recently, zanubrutinib was shown to be more effective than bendamustine plus rituximab as frontline therapy (SEQUOIA) [13], and as effective as ibrutinib but with a lower rate of AF (although not HTN) in relapsed/refractory CLL (ALPINE) [14].

The mechanisms underlying BTKi‐induced cardiotoxicity are not entirely known. Preclinical studies indicate that unintended inhibition of the PI3K‐Akt signaling pathway, specifically through Tec protein tyrosine kinase or C‐terminal Src kinase (CSK), might be a contributing factor [15, 16]. Additionally, ibrutinib suppresses human epidermal growth factor receptor 2 (HER2), a key component in maintaining the homeostasis of cardiac myocytes [17, 18]. Ibrutinib irreversibly binds to Src kinase and several non‐BTK kinases with similar cysteine residues at low nanomolar concentrations, a characteristic not exhibited by the BTK inhibitor acalabrutinib [19, 20]. This likely contributes to the differential adverse event profiles seen with first‐ and second‐generation BTKi.

Since clinical trial populations are highly selected due to the application of inclusion and exclusion criteria, the true rate of cardiotoxicity associated with first‐ and second‐generation BTKi and their impact on survival in unselected CLL patients is unclear. To address this knowledge gap, we applied propensity score matching (PSM) to CLL patients in the TriNetX Global Collaborative Network database who received acalabrutinib versus ibrutinib and compared the two cohorts for cardiovascular events and all‐cause mortality.

## 2 Methods

### 2.1 TriNetX Platform

This retrospective observational study utilized the TriNetX platform, a global federated health research network providing access to electronic medical records (EMRs) from various healthcare organizations, including academic medical centers and community hospitals. The dataset includes demographic information, diagnoses coded using the International Classification of Diseases, Ninth and Tenth Revisions, Clinical Modification (ICD‐10‐CM), and medication details from nearly 70 million individuals, mostly in the United States. Further information is available online (https://open.trinetx.com/company‐overview/).

### 2.2 Patient Selection

Inclusion criteria were: aged 18 years or older; diagnosis of CLL (ICD‐10‐CM code C91.1) or small cell B‐cell lymphoma (ICD‐10‐CM code C83.0); and treatment with ibrutinib (NLM:RXNORM:1442981) or acalabrutinib (NLM:RXNORM:1986808). Exclusion criteria were a previous history of HTN (ICD‐10‐CM code I10) or AF/flutter (ICD‐10‐CM code I48) in any treatment groups. Moreover, this study complies with the RECORD‐PE reporting guidelines [21], and the checklist is available in Table S2.

### 2.3 Propensity Score Matching

PSM was performed to balance the cohorts and thereby minimize the effect of confounding variables. Variables applied were age, sex, ethnicity, and comorbid conditions. Following the matching process, standardized differences were computed to evaluate the balance of baseline characteristics between the matched groups. A standardized difference below 0.1 was to indicate a negligible difference, implying that the groups were appropriately balanced.

### 2.4 Outcome Measurements

Assessed outcomes were new‐onset AF/flutter and HTN. Additionally, reported heart failure, ventricular arrhythmias, bleeding, and all‐cause mortality. The index event was the first reported treatment with either ibrutinib or acalabrutinib. Outcomes were measured within a 1095‐day time window starting from the index event.

### 2.5 Statistical Analyses

All statistical analyses were performed using the integrated statistical tools within the TriNetX platform, which included the R survival package v3.2‐3. Baseline characteristics were compared using the chi‐squared test for categorical variables and the independent‐sample t‐test for continuous variables. Longitudinal outcomes were analyzed using the Kaplan–Meier method, while hazard ratios (HRs) and 95% confidence intervals (CI) were estimated using the Cox proportional hazard model. All tests were two‐tailed, and *p*‐values were considered statistically significant at ≤ 0.05.

## 3 Results

### 3.1 Patient Characteristics

**FIGURE 1:**
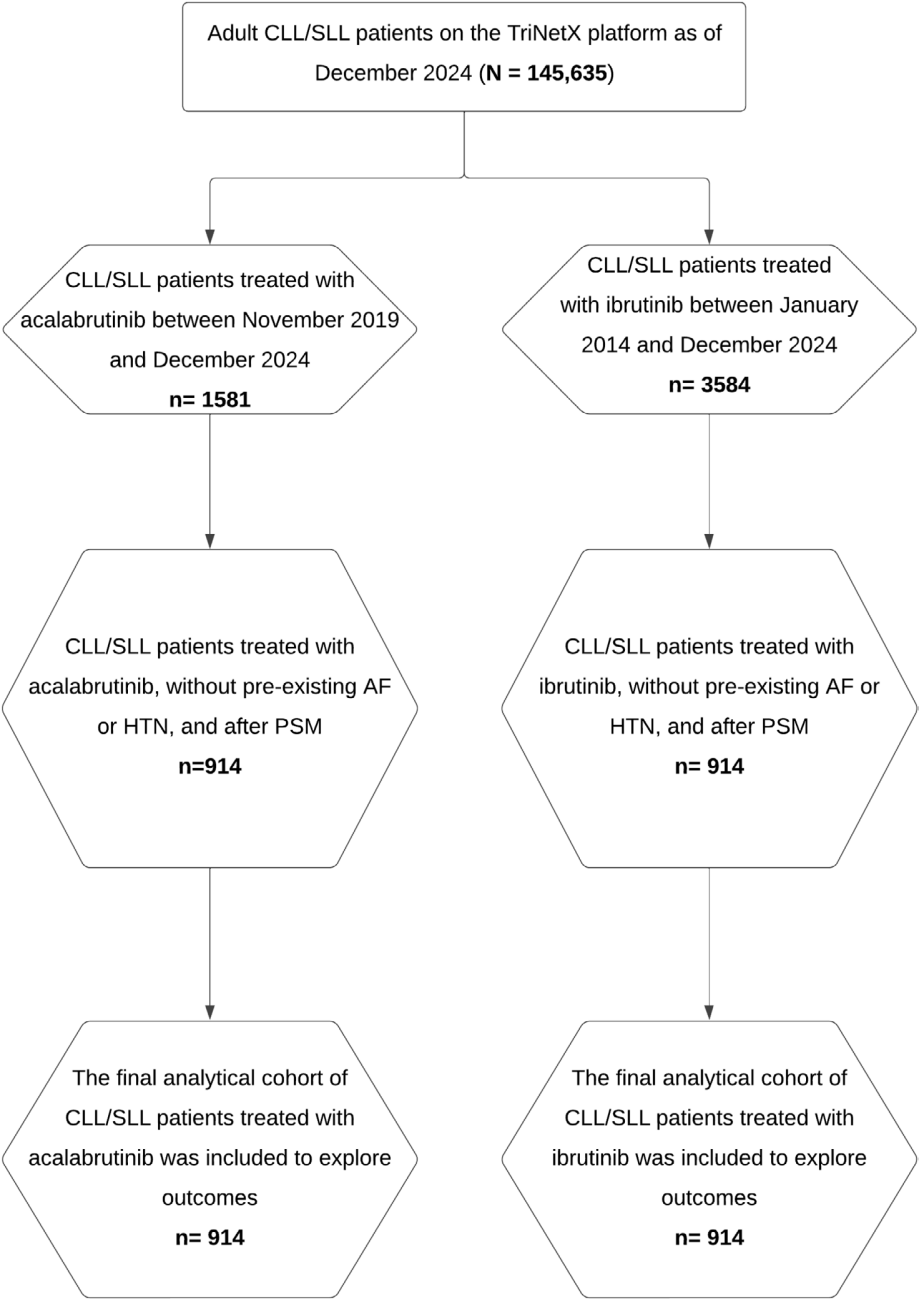
Flow diagram of acalabrutinib and ibrutinib cohorts. AF, atrial fibrillation; CLL, chronic lymphocytic leukemia; HTN, hypertension; PSM, propensity score matching; SLL, small lymphocytic leukemia.

The initial search found 3584 ibrutinib‐treated patients and 1581 who received acalabrutinib. After applying exclusion criteria and PSM to balance for baseline characteristics, medications, and co‐morbidity, each cohort comprised 914 patients (Figure 1, Table 1). The final ibrutinib and acalabrutinib cohorts were well balanced for demographics (age, sex, ethnicity) CLL‐related features (hepatosplenomegaly, prior antineoplastic chemotherapy), cardiovascular and cerebrovascular diseases (heart failure, ischemic heart diseases, cerebral infarction, and pulmonary embolism), risk factors for bleeding (abnormal coagulation profile) and cardiovascular disease (nicotine dependence, diabetes mellitus, obesity, lipid disorders, and chronic kidney disease), cardiovascular procedures, and relevant medication (alpha blockers, ACE inhibitors, beta blockers, calcium channel blockers, diuretics, antilipemic agents, anticoagulants, and platelet aggregation inhibitors); additional details are provided in Table S3.

**TABLE 1:** Baseline characteristics, medications, and diagnoses before and after propensity score matching (PSM).

Characteristic	Before PSM acalabrutinib (*N* = 920)	Before PSM ibrutinib (*N* = 2475)	Standardized difference before PSM	After PSM acalabrutinib (*N* = 914)	After PSM ibrutinib (*N* = 914)	Standardized difference after PSM
Age at index	68.1 ± 10.8	65.8 ± 10.1	0.223	68.0 ± 10.8	67.8 ± 9.7	0.025
Male (%)	581 (63.2%)	1626 (65.7%)	0.052	577 (63.1%)	573 (62.7%)	0.009
Female (%)	339 (36.8%)	850 (34.3%)	0.052	337 (36.9%)	341 (37.3%)	0.009
White (%)	706 (76.7%)	1865 (75.4%)	0.032	702 (76.8%)	731 (80.0%)	0.077
Black or African American (%)	43 (4.7%)	99 (4.0%)	0.033	41 (4.5%)	36 (3.9%)	0.027
Hepatomegaly and splenomegaly (%)	132 (14.3%)	316 (12.8%)	0.046	131 (14.3%)	126 (13.8%)	0.016
Abnormal coagulation profile (%)	0 (0.0%)	10 (0.4%)	0.090	0 (0.0%)	0 (0.0%)	—
Personal history of nicotine dependence (%)	53 (5.8%)	115 (4.6%)	0.050	52 (5.7%)	51 (5.6%)	0.005
Personal history of antineoplastic chemotherapy (%)	27 (2.9%)	89 (3.6%)	0.037	27 (3.0%)	29 (3.2%)	0.013
Heart failure (%)	16 (1.7%)	25 (1.0%)	0.063	13 (1.4%)	12 (1.3%)	0.009
Diabetes mellitus (%)	52 (5.7%)	136 (5.5%)	0.007	51 (5.6%)	38 (4.2%)	0.066
Hypertensive diseases (%)	0 (0.0%)	0 (0.0%)	—	0 (0.0%)	0 (0.0%)	—
AF/flutter (%)	0 (0.0%)	0 (0.0%)	—	0 (0.0%)	0 (0.0%)	—
Overweight, obesity (%)	30 (3.3%)	82 (3.3%)	0.003	30 (3.3%)	21 (2.3%)	0.060
Disorders of lipoprotein metabolism (%)	145 (15.8%)	395 (16.0%)	0.005	144 (15.8%)	133 (14.6%)	0.034
Chronic kidney disease (%)	45 (4.9%)	89 (3.6%)	0.064	44 (4.8%)	49 (5.4%)	0.025
Ischemic heart diseases (%)	52 (5.7%)	126 (5.1%)	0.025	50 (5.5%)	36 (3.9%)	0.072
Cerebral infarction (%)	10 (1.1%)	16 (0.6%)	0.048	10 (1.1%)	10 (1.1%)	< 0.001
Pulmonary embolism (%)	12 (1.3%)	33 (1.3%)	0.003	12 (1.3%)	10 (1.1%)	0.020
Surgical procedures on CV system (%)	401 (43.6%)	1109 (44.8%)	0.025	400 (43.8%)	408 (44.6%)	0.018
Alpha‐blockers (%)	77 (8.4%)	165 (6.7%)	0.065	74 (8.1%)	71 (7.8%)	0.012
ACE inhibitors (%)	57 (6.2%)	135 (5.5%)	0.032	56 (6.1%)	49 (5.4%)	0.033
Beta‐blockers (%)	77 (8.4%)	212 (8.6%)	0.007	77 (8.4%)	66 (7.2%)	0.045
Calcium channel blockers (%)	51 (5.5%)	85 (3.4%)	0.102	49 (5.4%)	50 (5.5%)	0.005
Diuretics (%)	84 (9.1%)	217 (8.8%)	0.013	84 (9.2%)	73 (8.0%)	0.043
Antilipemic agents (%)	185 (20.1%)	436 (17.6%)	0.064	183 (20%)	160 (17.5%)	0.064
Anticoagulants (%)	160 (17.4%)	401 (16.2%)	0.032	158 (17.3%)	143 (15.6%)	0.044
Platelet aggregation inhibitors (%)	117 (12.7%)	323 (13.1%)	0.010	117 (12.8%)	95 (10.4%)	0.075

### 3.2 Cardiovascular Outcomes

At 3 years after the index event, new‐onset AF/flutter occurred in 5.8% versus 11.7% [HR 0.59 (95% CI 0.43–0.83); *p* = 0.002] of patients treated with acalabrutinib and ibrutinib, respectively. New‐onset HTN also occurred significantly less often in patients treated with acalabrutinib [15% vs. 26.3%; HR 0.65 (95% CI 0.53–0.81); *p* < 0.05]. In contrast, there were no significant differences between the acalabrutinib and ibrutinib groups in the reported incidence of heart failure [4.6% vs. 5%; HR 1.13 (95% CI 0.74–1.73); *p* = 0.5] or ventricular arrhythmias [1.1% vs. 1.6%; HR 0.65 (95% CI 0.27–1.56); *p* = 0.3] (Table 2).

**TABLE 2:** Comparative outcomes of acalabrutinib and ibrutinib cohorts.

Outcome	Cohort	Patients in cohort	Patients with outcome (%)	HR (95% CI)
AF/flutter	Acalabrutinib	914	53 (5.8%)	0.59 (0.43–0.83)
	Ibrutinib	914	107 (11.7%)	
Hypertension	Acalabrutinib	914	137 (15.0%)	0.65 (0.53–0.81)
	Ibrutinib	914	240 (26.3%)	
Heart failure	Acalabrutinib	914	42 (4.6%)	1.13 (0.74–1.73)
	Ibrutinib	914	46 (5.0%)	
Ventricular arrhythmias	Acalabrutinib	914	10 (1.1%)	0.65 (0.27–1.56)
	Ibrutinib	914	15 (1.6%)	
Bleeding	Acalabrutinib	914	26 (2.8%)	0.76 (0.46–1.25)
	Ibrutinib	914	43 (4.7%)	
Mortality	Acalabrutinib	914	83 (9.0%)	0.98 (0.73–1.30)
	Ibrutinib	914	109 (11.9%)	

Abbreviations: AF, atrial fibrillation; CI, confidence interval; HR, hazard ratio.

### 3.3 Bleeding and All‐Cause Mortality

**FIGURE 2:**
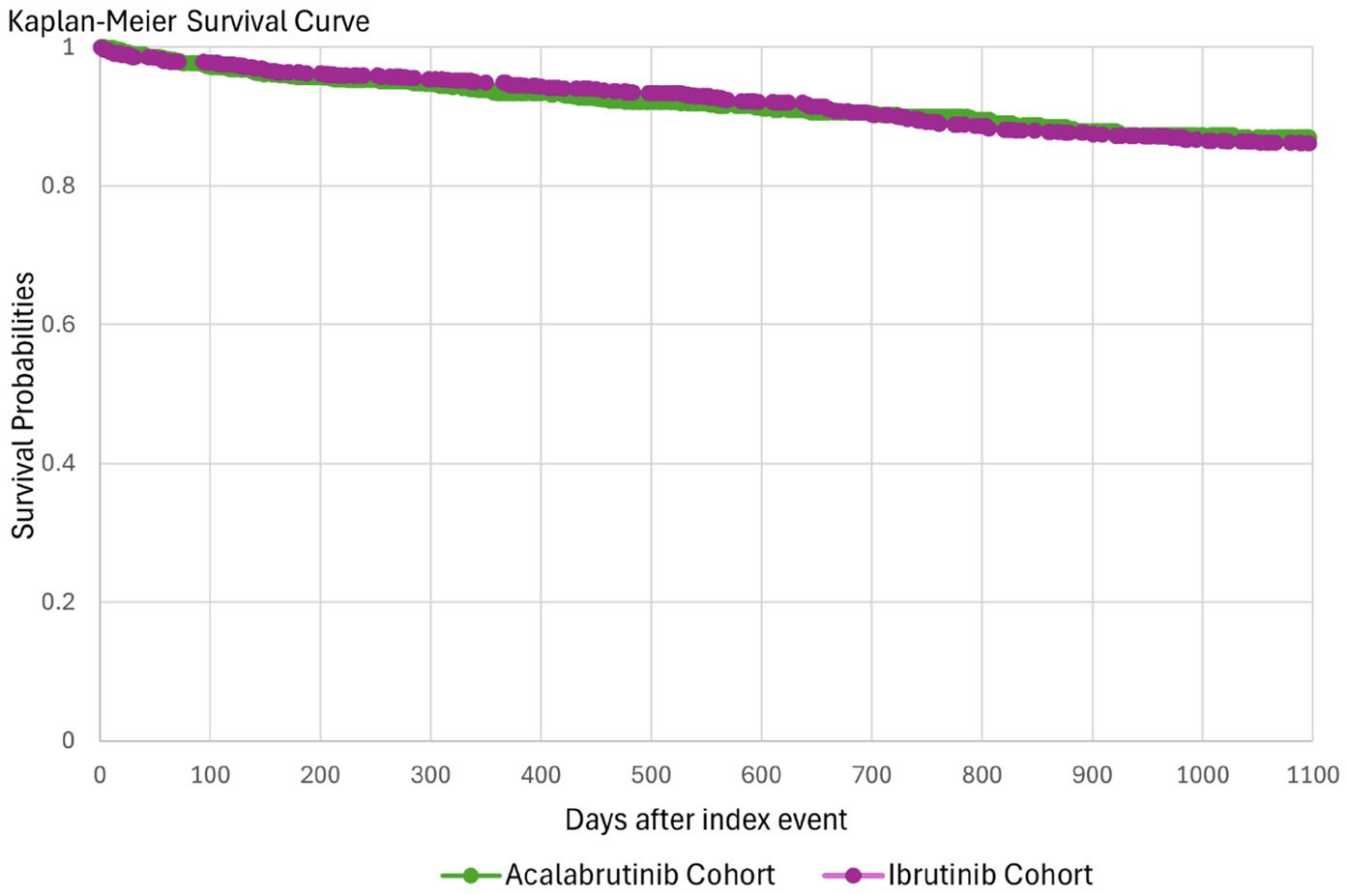
Kaplan–Meier survival analysis.

Bleeding events occurred in 2.8% versus 4.7% of patients treated with acalabrutinib and ibrutinib, respectively, with no statistical significance observed [HR 0.76 (95% CI 0.46–1.25); *p* = 0.2]. The all‐cause mortality was not statistically significant in patients treated with acalabrutinib compared to ibrutinib [11.9 vs. 9%; HR 0.98 (95% CI 0.73–1.30); *p* = 0.8] (Table 2, Figure 2).

## 4 Discussion

This study provides new insights into the comparative safety profiles of ibrutinib and acalabrutinib in patients with CLL/SLL, focusing on cardiovascular outcomes, bleeding events, and all‐cause mortality. By utilizing a large population‐level dataset, our study showed that acalabrutinib produces less AF and HTN compared to ibrutinib; in doing so, it confirms the findings of the ELEVATE‐RR trial in a real‐world setting.

The incidence of AF in the present study was significantly lower in patients treated with acalabrutinib (5.8%) compared to those who received ibrutinib (11.7%). In the ELEVATE‐RR trial, the incidence of AF in the acalabrutinib arm was also significantly lower than in the ibrutinib arm (9.4% vs. 16.0%; *p* = 0.02) with a 48% reduction in cumulative risk [12]. Recent evidence from the phase III ALPINE study comparing zanubrutinib with ibrutinib has shown a similar effect [14], and it seems likely that these differential effects are due to off‐target effects of ibrutinib that are not shared by more selective BTKis. Of potential relevance here is that ibrutinib, but not acalabrutinib [19, 20] or zanubrutinib [22, 23], binds to Src and related kinases at low‐nanomolar concentrations. Atrial myocytes are distinctive for their high expression of CSK, a key regulator of Src‐family kinases (SFK), and inhibition of CSK has been shown to play a key role in ibrutinib‐induced AF in an animal model [16]. Ibrutinib also inhibits HER2 which contributes to the maintenance of cardiac myocyte homeostasis [17, 24]. Since AF is the most common toxicity resulting in treatment cessation or interruption [25, 26], more selective BTKis with similar efficacy and a lower risk of AF offer a significant advantage over ibrutinib. Irrespective of which BTKi is used, management of BTKi‐induced AF requires careful weighing up of the risk of AF‐induced thromboembolism [7, 27] versus the risk of bleeding due to the combined effects of anticoagulation and ongoing BTKi therapy [28]. The selection of non‐vitamin K antagonist oral anticoagulants is recommended unless specific contraindications exist [27].

The incidence of HTN in the present study was significantly lower in patients treated with acalabrutinib (15%) compared to those who received ibrutinib (26.3%). These findings are entirely consistent with the ELEVATE‐RR trial, where HTN rates for acalabrutinib and ibrutinib were 9% versus 23%, respectively, for all grades and 4% versus 9%, respectively, for grade 3 or higher [12]. Interestingly, in the ALPINE trial, which compared ibrutinib with the more selective second‐generation BTKi, zanubrutinib, the rate of new‐onset HTN was similar in the two treatment arms, whereas grade 3 HTN occurred slightly more frequently in the zanubrutinib arm (14.8% and 11.1%) [14]. In contrast, in the ASPEN trial, which compared ibrutinib with zanubrutinib in patients with Waldenstrom's macroglobulinemia (WM), a lower incidence of HTN was observed in the zanubrutinib group but not until at least 12 months of follow‐up [29]. The true relative risk of HTN in CLL patients receiving zanubrutinib compared to ibrutinib is therefore unclear at present.

The incidence of heart failure in the present study was similar in the acalabrutinib and ibrutinib cohorts (4.6% vs. 5%), respectively. This finding is entirely consistent with the ELEVATE‐RR trial, which also reported a similar incidence of heart failure across both treatment arms [12]. This, together with the established link between long‐term (> 3 years) treatment with ibrutinib and heart failure [5, 7, 17, 30], suggests that long‐term treatment with newer BTKi may also be associated with an increased risk of heart failure.

Similar considerations apply to ventricular arrhythmias, which occurred in 1.1% versus 1.6% of patients, who were treated with acalabrutinib and ibrutinib, respectively, in this study. Guha et al. [31] found that ibrutinib therapy substantially increases the risk of ventricular arrhythmias in patients with hematological malignancies, with an incidence rate of 596 per 100 000 person‐years during ibrutinib exposure compared to 48.1 cases per 100 000 person‐years in untreated patients. Acalabrutinib has also been linked to ventricular arrhythmias and sudden death, with a median time to onset of more than 1 year [32]. Murine studies suggest that susceptibility to ventricular arrhythmias is increased by both ibrutinib and acalabrutinib, with similar effects on calcium handling in cultured myocytes treated with insulin‐like growth factor 1, including a reduction in calcium transient amplitude and a slower decay of calcium transients [7, 33].

The proportion of patients reported to have experienced bleeding events in the present study was 2.8% versus 4.7% in the acalabrutinib and ibrutinib cohorts, respectively, compared to corresponding values in the ELEVATE‐RR trial of 38% versus 51% for all bleeding events and 4.5% versus 5.3% for major bleeding events [12]. This suggests that only major bleeding events were captured in the present study (Table S1). Assuming this to be the case, the results of the present study are entirely in keeping with those of the ELEVATE‐RR trial. Bleeding is a well‐established complication of ibrutinib, with one study showing a 4.9‐fold increase in the risk of bleeding of any severity and a 7.5‐fold increase in the risk of major bleeding compared with non‐BTKi therapies [28]. BTK is expressed in platelets, where it mediates collagen signaling via GPVI [34]. Therefore, the increased bleeding associated with ibrutinib compared with non‐BTK therapies is likely to reflect an on‐target effect. In keeping with this, the incidence of minor and major bleeding episodes was similar for ibrutinib and zanubrutinib in the ALPINE study [14]. On the other hand, the higher risk of any‐grade bleeding seen with ibrutinib compared with acalabrutinib or zanubrutinib in the ELEVATE RR and ASPEN trials [12, 29], could potentially be explained by off‐target effects in platelets such as inhibition of SFK or BTK homologues TEC and BMX [35], differential effects on the conformational state of BTK [36], or increased anticoagulant usage due to a higher rate of AF/flutter in patients receiving ibrutinib compared to the more selective BTK inhibitors [37].

Despite clear differences in AF and HTN, all‐cause mortality in the present study was similar for the acalabrutinib and ibrutinib cohorts [9% vs. 11.9%; HR 0.98 (95% CI 0.73–1.30; *p* = 0.8)]. Although the underlying causes of mortality are unknown within the database, populations in real‐world studies have shown a higher prevalence of comorbidities and increased frailty [38]. This is consistent with the ELEVATE‐RR trial, where the rate of treatment discontinuation due to cardiac events was over five times higher among patients treated with ibrutinib compared to acalabrutinib [12] and suggests that cardiovascular events, although undesirable, are nevertheless manageable in most patients and that further, effective CLL therapy is available.

Our study has several limitations. These include its retrospective design, which can introduce selection and other biases; the PSM analysis, which does not consider unmeasured confounding factors such as CLL prognostic factors and compliance with BTKi therapy; the study's confinement to the TriNetX network, which potentially limits its general applicability; the exclusion of patients with pre‐existing HTN and AF/flutter, which limits the study's scope; and incomplete data or coding errors, which limit the study's reliability. Additionally, the severity of each comorbidity recorded in the database is not specified, although the similarity between the two cohorts in outcomes other than AF/flutter and HTN provides indirect evidence that relevant comorbidities were well‐matched. Despite these limitations, our study nevertheless provides new evidence that acalabrutinib has a favorable cardiovascular toxicity profile compared to ibrutinib in a real‐world setting. In doing so, it validates findings from the ELEVATE‐RR trial and adds weight to the idea that acalabrutinib is a safer alternative to ibrutinib, especially for older patients [1, 39] and those with other risk factors for ibrutinib‐induced cardiotoxicity, such as increased atrial diameter, male sex, and arterial HTN [40, 41].

In conclusion, our study highlights the importance of considering BTKi‐related cardiovascular and bleeding risks when considering therapy options for individual patients, as well as the need to develop new and better approaches for assessing and minimizing these risks, detecting cardiovascular toxicity at an early stage, and ameliorating such toxicity when it occurs.

## Author Contributions

A.M. designed the study, conducted the statistical analyses, and drafted the manuscript. Y.X.G., A.S., S.W., and C.T.S. contributed to the study design discussion and provided critical review, revisions, and enhancements to improve manuscript quality. As joint senior authors, G.Y.H.L. and A.R.P. provided supervision throughout the study, including comprehensive review, editorial contributions, and final quality improvements to the manuscript. All authors have reviewed the final version of the manuscript and have agreed to its submission.

## Ethics Statement

This study was conducted according to the guidelines of the Declaration of Helsinki, and no approval was needed since data was de‐identified.

## Consent

The authors have nothing to report.

## Conflicts of Interest

The authors declare no conflicts of interest.

## Supporting information


Data S1.

## Data Availability

Additional information about the statistical analyses and propensity score matching can be found in the supplementary data.
