# Supplementary material for: A Comparative Analysis of Cardiovascular Events Associated With Acalabrutinib Versus Ibrutinib in Chronic Lymphocytic Leukemia: Insights From a Global Federated Network
Source: Pharmacol Res Perspect. 2025 May 7;13(3):e70113. doi: 10.1002/prp2.70113 (PMC12059286; doi:10.1002/prp2.70113)
Supplement: Supplementary file 1 — Data S1. [file PRP2-13-e70113-s001.pdf]

A Comparative Analysis of Cardiovascular Events Associated with Acalabrutinib Versus Ibrutinib in Chronic Lymphocytic Leukemia: Insights from a Global Federated Network

Abdulrahman Majrashi, Ying X. Gue, Alena Shantsila, Stella Williams, Catrin Tudur Smith, Gregory Y. H. Lip, Andrew R. Pettitt

Supplementary Data

The TriNetX database compiles data from member healthcare organizations (HCOs), which is sourced primarily from their electronic health records (EHR) systems. A typical HCO is a large academic health institution, and its data typically include contributions from its affiliated hospitals and clinics. Frequently, the HCO consists of multiple facilities, such as central hospitals and satellite sites. The data are stored on TriNetX either through a physical server located in the institution's data centre or via a virtual appliance hosted elsewhere. The TriNetX platform consists of a network of these appliances, forming a federated system. This system can disseminate queries across the network, and the resulting data are gathered and aggregated. Once the data are shared with the network, they are standardised according to controlled clinical terminologies and undergo a quality assurance process, which includes data cleaning that excludes records not meeting TriNetX quality criteria. Each data refresh is subject to a rigorous internal quality review, assessing aspects such as conformity, completeness, and plausibility (http://doi.org/10.13063/2327-9214.1244). The platform ensures compliance with the Health Insurance Portability and Accountability Act (HIPAA) through deidentification of patient data. The available data types within the network include demographics, diagnoses (ICD-10-CM codes), procedures (ICD-10-PCS or CPT codes), and clinical measurements (LOINC codes). While comprehensive data on diagnoses and procedures are available, other variables such as socioeconomic status and lifetime health factors are less thoroughly represented. A notable advantage of EHR data over insurance claims data is that it includes information on both patients with and without insurance. Additionally, EHR data, compared to survey data, provide more accurate reflections of the diagnostic rates among populations looking for healthcare services, offering more clearer picture of the burden of specific conditions on healthcare systems. However, a significant limitation of using diagnoses is the exclusion of individuals with undiagnosed conditions who have not yet been examined and sought by medical care providers. Another limitation of EHR data is that if a patient receives care from multiple HCOs, and one of these organisations is not part

of the TriNetX network, some of the patient's medical history may not be reachable and accessible. Although the federated network of healthcare organisations mitigates this issue, but it cannot fully eliminate it.

In propensity score-matched analyses, TriNetX uses logistic regression from the scikit-learn package in Python (version 3.7) to perform 1:1 greedy nearest neighbour matching with a calliper of 0.1 pooled standard deviations. To prevent bias from the nearest neighbour algorithm, the data rows are randomised before matching. Baseline characteristics are considered well matched if the standardised mean difference between cohorts is less than 0.1.

(https://www.tandfonline.com/doi/full/10.1080/00273171.2011.568786).

Supplementary Table 1. ICD-10-CM codes for primary and secondary outcomes.

**Outcomes**	**ICD-10-CM-codes**
Atrial fibrillation	I48 Atrial fibrillation and flutter
Hypertension	I10-I1A Hypertensive disease
Acute Heart failure	I50.21 Acute Systolic heart failure<br>I50.23 Acute on chronic systolic heart failure<br>I50.31 Acute diastolic heart failure<br>I50.33 Acute on chronic diastolic heart failure
Ventricular arrhythmias	I49.0 Ventricular fibrillation and flutter<br>and/or<br>I47.2 Ventricular tachycardia
Bleeding	R58 Hemorrhage, not elsewhere classified<br>and/ or<br>I60 Nontraumatic subarachnoid hemorrhage<br>I61 Nontraumatic intracerebral hemorrhage<br>I62 Other and unspecified Nontraumatic intracranial hemorrhage

	K92.2 Gastrointestinal hemorrhage, unspecified
All-cause death	Deceased (variable codified by TriNetX).

Supplementary table 2 The RECORD statement for pharmacoepidemiology (RECORD-PE) checklist of items, extended from the STROBE and RECORD statements, which should be reported in non-interventional pharmacoepidemiological studies using routinely collected health data

Item No	STROBE items	RECORD items	RECORD-PE items	Page No
**Title and abstract**				
1	(a) Indicate the study's design with a commonly used term in the title or the abstract.<br>(b) Provide in the abstract an informative and balanced summary of what was done and what was found.	1.1: The type of data used should be specified in the title or abstract. When possible, the name of the databases used should be included.<br>1.2: If applicable, the geographical region and timeframe within which the study took place should be reported in the title or abstract.<br>1.3: If linkage between databases was conducted for the study, this should be clearly stated in the title or abstract.	—	1,2
**Introduction**				
Background rationale				
2	Explain the scientific background and rationale for the investigation being reported.	—	—	3,4
Objectives				
3	State specific objectives, including any prespecified hypotheses.	—	—	4
**Methods**				
Study design				
4	Present key elements of study design early in the paper.	—	4.a: Include details of the specific study design (and its features) and report the use of multiple designs if used.<br>4.b: The use of a diagram(s) is recommended to illustrate key aspects of the study design(s), including exposure, washout, lag and observation periods, and covariate definitions as relevant.	5,6
Setting				
5	Describe the setting, locations, and relevant dates, including periods of recruitment, exposure, follow-up, and data collection.	—	—	5,6
Participants				
6	(a) Cohort study—give the eligibility criteria, and the sources and methods of selection of participants. Describe methods of follow-up. Case-control study—give the eligibility criteria, and the sources and methods of case ascertainment and control selection. Give the rationale for the choice of cases and controls. Cross sectional study—give the eligibility criteria, and the sources and methods of selection of participants.<br>(b) Cohort study—for matched studies, give matching criteria and number of exposed and unexposed. Case-control study—for matched studies, give matching criteria and the number of controls per case.	6.1: The methods of study population selection (such as codes or algorithms used to identify participants) should be listed in detail. If this is not possible, an explanation should be provided.<br>6.2: Any validation studies of the codes or algorithms used to select the population should be referenced. If validation was conducted for this study and not published elsewhere, detailed methods and results should be provided.<br>6.3: If the study involved linkage of databases, consider use of a flow diagram or other graphical display to demonstrate the data linkage process, including the number of individuals with linked data at each stage.	6.1.a: Describe the study entry criteria and the order in which these criteria were applied to identify the study population. Specify whether only users with a specific indication were included and whether patients were allowed to enter the study population once or if multiple entries were permitted. See explanatory document for guidance related to matched designs.	5,6
Variables				
7	Clearly define all outcomes, exposures, predictors, potential confounders, and effect modifiers. Give diagnostic criteria, if applicable.	7.1: A complete list of codes and algorithms used to classify exposures, outcomes, confounders, and effect modifiers should be provided. If these cannot be reported, an explanation should be provided.	7.1.a: Describe how the drug exposure definition was developed.<br>7.1.b: Specify the data sources from which drug exposure information for individuals was obtained.<br>7.1.c: Describe the time window(s) during which an individual is considered exposed to the drug(s). The rationale for selecting a particular time window should be provided. The extent of potential left truncation or left censoring should be specified.<br>7.1.d: Justify how events are attributed to current, prior, ever, or cumulative drug exposure.<br>7.1.e: When examining drug dose and risk attribution, describe how current, historical or time on therapy are considered.<br>7.1.f: Use of any comparator groups should be outlined and justified.<br>7.1.g: Outline the approach used to handle individuals with more than one relevant drug exposure during the study period.	5,6
Data sources/measurement				
8	For each variable of interest, give sources of data and details of methods of assessment (measurement). Describe comparability of assessment methods if there is more than one group.	—	8.a: Describe the healthcare system and mechanisms for generating the drug exposure records. Specify the care setting in which the drug(s) of interest was prescribed.	5,6

(*Continued*)

Supplementary table 2 (*Continued*)

Item No	STROBE items	RECORD items	RECORD-PE items	Page No
Bias				
9	Describe any efforts to address potential sources of bias.	—	—	5,19
Study size				
10	Explain how the study size was arrived at.	—	—	6,21
Quantitative variables				
11	Explain how quantitative variables were handled in the analyses. If applicable, describe which groupings were chosen, and why.	—	—	6
Statistical methods				
12	(a) Describe all statistical methods, including those used to control for confounding.<br>(b) Describe any methods used to examine subgroups and interactions.<br>(c) Explain how missing data were addressed.<br>(d) Cohort study—if applicable, explain how loss to follow-up was addressed. Case-control study—if applicable, explain how matching of cases and controls was addressed. Cross sectional study—if applicable, describe analytical methods taking account of sampling strategy.<br>(e) Describe any sensitivity analyses.	—	12.1.a: Describe the methods used to evaluate whether the assumptions have been met.<br>12.1.b: Describe and justify the use of multiple designs, design features, or analytical approaches.	6
Data access and cleaning methods				
12	—	12.1: Authors should describe the extent to which the investigators had access to the database population used to create the study population.<br>12.2: Authors should provide information on the data cleaning methods used in the study.	—	5,6
Linkage				
12	—	12.3: State whether the study included person level, institutional level, or other data linkage across two or more databases. The methods of linkage and methods of linkage quality evaluation should be provided.	—	5,6
**Results**				
Participants				
13	(a) Report the numbers of individuals at each stage of the study (eg, numbers potentially eligible, examined for eligibility, confirmed eligible, included in the study, completing follow-up, and analysed).<br>(b) Give reasons for non-participation at each stage.<br>(c) Consider use of a flow diagram.	13.1: Describe in detail the selection of the individuals included in the study (that is, study population selection) including filtering based on data quality, data availability, and linkage. The selection of included individuals can be described in the text or by means of the study flow diagram.	—	6,7
Descriptive data				
14	(a) Give characteristics of study participants (eg, demographic, clinical, social) and information on exposures and potential confounders.<br>(b) Indicate the number of participants with missing data for each variable of interest.<br>(c) Cohort study—summarise follow-up time (eg, average and total amount).	—	—	6,7,18,19
Outcome data				
15	Cohort study—report numbers of outcome events or summary measures over time. Case-control study—report numbers in each exposure category, or summary measures of exposure. Cross sectional study—report numbers of outcome events or summary measures.	—	—	7,20
Main results				
16	(a) Give unadjusted estimates and, if applicable, confounder adjusted estimates and their precision (eg, 95% confidence intervals). Make clear which confounders were adjusted for and why they were included.<br>(b) Report category boundaries when continuous variables are categorised.<br>(c) If relevant, consider translating estimates of relative risk into absolute risk for a meaningful time period.	—	—	7,20

Supplementary table 2 (*Continued*)				
**Item No**	**STROBE items**	**RECORD items**	**RECORD-PE items**	**Page No**
Other analyses				
17	Report other analyses done—eg, analyses of subgroups and interactions, and sensitivity analyses.	—	—	-
**Discussion**				
Key results				
18	Summarise key results with reference to study objectives.	—	—	6,7,19,20
Limitations				
19	Discuss limitations of the study, taking into account sources of potential bias or imprecision. Discuss both direction and magnitude of any potential bias.	19.1: Discuss the implications of using data that were not created or collected to answer the specific research question(s). Include discussion of misclassification bias, unmeasured confounding, missing data, and changing eligibility over time, as they pertain to the study being reported.	19.1.a: Describe the degree to which the chosen database(s) adequately captures the drug exposure(s) of interest.	12
Interpretation				
20	Give a cautious overall interpretation of results considering objectives, limitations, multiplicity of analyses, results from similar studies, and other relevant evidence.	—	20.a: Discuss the potential for confounding by indication, contraindication or disease severity or selection bias (healthy adherer/sick stopper) as alternative explanations for the study findings when relevant.	8,9,10,11
Generalisability				
21	Discuss the generalisability (external validity) of the study results.	—	—	12
**Other information**				
Funding				
22	Give the source of funding and the role of the funders for the present study and, if applicable, for the original study on which the present article is based.	—	—	13
Accessibility of protocol, raw data, and programming code				
22	—	22.1: Authors should provide information on how to access any supplemental information such as the study protocol, raw data, or programming code.	—	13

RECORD=reporting of studies conducted using observational routinely collected data; RECORD-PE=RECORD for pharmacoepidemiological research; STROBE=strengthening the reporting of observational studies in epidemiology. This checklist has been duplicated from table 1 in *BMJ* 2018;363:k3532, as a standalone document for readers to print out or fill in electronically.

Supplementary Table 3. Baseline characteristics for two cohorts before and after propensity score matching.

**Cohort 1 and cohort 2 patient count before and after propensity score matching**		
Cohort	Patient count before matching	Patient count after matching
1 - Acalabrutinib 17	920	914
2 - Ibrutinib 17	2,475	914

Propensity score density function - Before and after matching (cohort 1 - purple, cohort 2 - green)

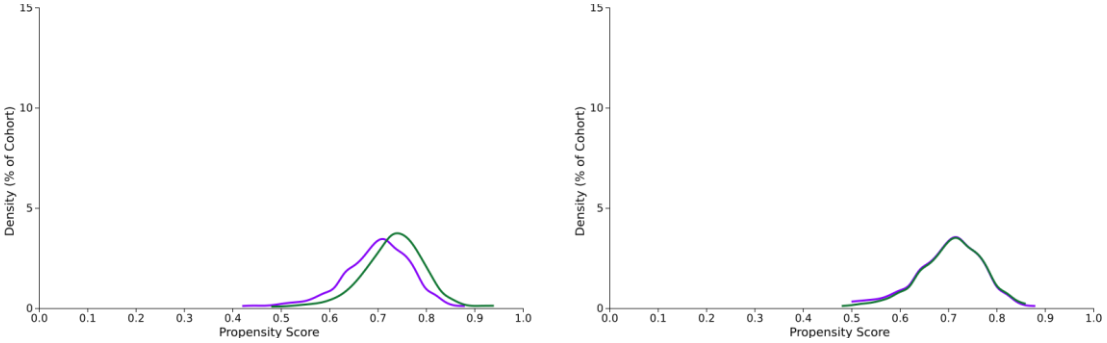

Cohort 1 (N = 920) and cohort 2 (N = 2,475) characteristics before propensity score matching

Demographics

Cohort			Mean ± SD	Patients	% of Cohort	P-Value	Std diff.
1<br>2	AI	Age at Index	68.1 +/- 10.8<br>65.8 +/- 10.1	920<br>2,475	100%<br>100%	<0.001	0.223
1<br>2	2106-3	White		706<br>1,865	76.7%<br>75.4%	0.403	0.032
1<br>2	F	Female		339<br>850	36.8%<br>34.3%	0.174	0.052
1<br>2	2054-5	Black or African American		43<br>99	4.7%<br>4%	0.383	0.033

Diagnosis

Cohort			Mean ± SD	Patients	% of Cohort	P-Value	Std diff.
1<br>2	R16	Hepatomegaly and splenomegaly, not elsewhere classified		132<br>316	14.3%<br>12.8%	0.227	0.046
1<br>2	R79.1	Abnormal coagulation profile		0<br>10	0%<br>0.4%	0.054	0.090
1<br>2	Z87.891	Personal history of nicotine dependence		53<br>115	5.8%<br>4.6%	0.183	0.050
1<br>2	Z92.21	Personal history of antineoplastic chemotherapy		27<br>89	2.9%<br>3.6%	0.346	0.037
1<br>2	I50	Heart failure		16<br>25	1.7%<br>1.0%	0.084	0.063
1<br>2	E08-E13	Diabetes mellitus		52<br>136	5.7%<br>5.5%	0.859	0.007
1<br>2	I10-I1A	Hypertensive diseases		0<br>0	0%<br>0%	--	--
1<br>2	I48	Atrial fibrillation and flutter		0<br>0	0%<br>0%	--	--
1<br>2	E65-E68	Overweight, obesity and other hyperalimentation		30<br>82	3.3%<br>3.3%	0.940	0.003
1<br>2	E78	Disorders of lipoprotein metabolism and other lipidemias		145<br>395	15.8%<br>16.0%	0.888	0.005

Cohort			Mean ± SD	Patients	% of Cohort	P-Value	Std diff.
1<br>2	N18	Chronic kidney disease (CKD)		45<br>89	4.9%<br>3.6%	0.085	0.064
1<br>2	I20-I25	Ischemic heart diseases		52<br>126	5.7%<br>5.1%	0.514	0.025
1<br>2	I63	Cerebral infarction		10<br>16	1.1%<br>0.6%	0.191	0.048
1<br>2	I26	Pulmonary embolism		12<br>33	1.3%<br>1.3%	0.948	0.003
**Procedure**							
Cohort			Mean ± SD	Patients	% of Cohort	P-Value	Std diff.
1<br>2	1006056	Surgical Procedures on the Cardiovascular System		401<br>1,109	43.6%<br>44.8%	0.525	0.025
**Medication**							
Cohort			Mean ± SD	Patients	% of Cohort	P-Value	Std diff.
1<br>2	CV150	ALPHA BLOCKERS/RELATED		77<br>165	8.4%<br>6.7%	0.087	0.065
1<br>2	CV800	ACE INHIBITORS		57<br>135	6.2%<br>5.5%	0.406	0.032
1<br>2	CV100	BETA BLOCKERS/RELATED		77<br>212	8.4%<br>8.6%	0.856	0.007
1<br>2	CV200	CALCIUM CHANNEL BLOCKERS		51<br>85	5.5%<br>3.4%	0.005	0.102
1<br>2	CV700	DIURETICS		84<br>217	9.1%<br>8.8%	0.741	0.013
1<br>2	CV350	ANTILIPEMIC AGENTS		185<br>436	20.1%<br>17.6%	0.095	0.064
1<br>2	BL110	ANTICOAGULANTS		160<br>401	17.4%<br>16.2%	0.407	0.032
1<br>2	BL117	PLATELET AGGREGATION INHIBITORS		117<br>323	12.7%<br>13.1%	0.797	0.010
**Cohort 1 (N = 914) and cohort 2 (N = 914) characteristics after propensity score matching**							
**Demographics**							
Cohort			Mean ± SD	Patients	% of Cohort	P-Value	Std diff.
1<br>2	AI	Age at Index	68.0 +/- 10.8<br>67.8 +/- 9.7	914<br>914	100%<br>100%	0.595	0.025
1<br>2	2106-3	White		702<br>731	76.8%<br>80.0%	0.099	0.077
1<br>2	F	Female		337<br>341	36.9%<br>37.3%	0.846	0.009
1<br>2	2054-5	Black or African American		41<br>36	4.5%<br>3.9%	0.560	0.027
**Diagnosis**							
Cohort			Mean ± SD	Patients	% of Cohort	P-Value	Std diff.
1<br>2	R16	Hepatomegaly and splenomegaly, not elsewhere classified		131<br>126	14.3%<br>13.8%	0.737	0.016
1<br>2	R79.1	Abnormal coagulation profile		0<br>0	0%<br>0%	--	--
1<br>2	Z87.891	Personal history of nicotine dependence		52<br>51	5.7%<br>5.6%	0.919	0.005
1<br>2	Z92.21	Personal history of antineoplastic chemotherapy		27<br>29	3.0%<br>3.2%	0.786	0.013

Cohort	Code	Description	Mean ± SD	Patients	% of Cohort	P-Value	Std diff.
1<br>2	I50	Heart failure		13<br>12	1.4%<br>1.3%	0.840	0.009
1<br>2	E08-E13	Diabetes mellitus		51<br>38	5.6%<br>4.2%	0.158	0.066
1<br>2	I10-I1A	Hypertensive diseases		0<br>0	0%<br>0%	--	--
1<br>2	I48	Atrial fibrillation and flutter		0<br>0	0%<br>0%	--	--
1<br>2	E65-E68	Overweight, obesity and other hyperalimentation		30<br>21	3.3%<br>2.3%	0.201	0.060
1<br>2	E78	Disorders of lipoprotein metabolism and other lipidemias		144<br>133	15.8%<br>14.6%	0.473	0.034
1<br>2	N18	Chronic kidney disease (CKD)		44<br>49	4.8%<br>5.4%	0.595	0.025
1<br>2	I20-I25	Ischemic heart diseases		50<br>36	5.5%<br>3.9%	0.122	0.072
1<br>2	I63	Cerebral infarction		10<br>10	1.1%<br>1.1%	1	<0.001
1<br>2	I26	Pulmonary embolism		12<br>10	1.3%<br>1.1%	0.668	0.020
**Procedure**							
Cohort			Mean ± SD	Patients	% of Cohort	P-Value	Std diff.
1<br>2	1006056	Surgical Procedures on the Cardiovascular System		400<br>408	43.8%<br>44.6%	0.706	0.018
**Medication**							
Cohort			Mean ± SD	Patients	% of Cohort	P-Value	Std diff.
1<br>2	CV150	ALPHA BLOCKERS/RELATED		74<br>71	8.1%<br>7.8%	0.795	0.012
1<br>2	CV800	ACE INHIBITORS		56<br>49	6.1%<br>5.4%	0.482	0.033
1<br>2	CV100	BETA BLOCKERS/RELATED		77<br>66	8.4%<br>7.2%	0.338	0.045
1<br>2	CV200	CALCIUM CHANNEL BLOCKERS		49<br>50	5.4%<br>5.5%	0.918	0.005
1<br>2	CV700	DIURETICS		84<br>73	9.2%<br>8.0%	0.359	0.043
1<br>2	CV350	ANTILIPEMIC AGENTS		183<br>160	20.0%<br>17.5%	0.168	0.064
1<br>2	BL110	ANTICOAGULANTS		158<br>143	17.3%<br>15.6%	0.344	0.044
1<br>2	BL117	PLATELET AGGREGATION INHIBITORS		117<br>95	12.8%<br>10.4%	0.108	0.075
